# Society Meetings

**Published:** 1918-09

**Authors:** 


### SOCIETY MEETINGS

Brief reports and announcements of meetings of societies of Western New York, and those of general scope, are requested from Secretaries. Copy should be on hand the fifteenth of the month. Full transactions will be published at cost of composition.

---

**The Gross Medical Club** held their bi-monthly meeting at the Motor Boat Club House, Silver Creek, N. Y., Thursday, Aug. 15th, as the guest of Dr. O. B. Barber.

A most interesting and instructive address, Crime and the Human Race, was given by Mr. Henry J. Girvin, Chief of the Buffalo Department of Police.

Capt. and Surgeon T. J. Kee of the British Army also addressed the meeting giving some first hand information from an experience of over two years on the western front.

Dr. B. E. Smith the president acted as toastmaster at the dinner which was served later on the broad veranda of the club overlooking Lake Erie.

